# Optimizing biomass partitioning in wheat using UAV-based hyperspectral phenomic and genomic prediction: kernel-based and machine learning approaches

**DOI:** 10.3389/fpls.2026.1740337

**Published:** 2026-02-16

**Authors:** Sudip Kunwar, Md Ali Babar, Diego Jarquin, Yiannis Ampatzidis, Naeem Khan, Janam Prabhat Acharya, Jordan McBreen, Samuel Adewale, Gina Brown-Guedira

**Affiliations:** 1 Plant Breeding Graduate Program, University of Florida, Gainesville, FL, United States; 2 Department of Agronomy, University of Florida, Gainesville, FL, United States; 3 Agricultural and Biological Engineering Department, Southwest Florida Research and Education Center, University of Florida, Institute of Food and Agricultural Sciences (IFAS), Immorkalee, FL, United States; 4 Plant Science Research, United States Department of Agriculture- Agricultural Research Service Southeast Area (USDA-ARS SEA), Raleigh, NC, United States

**Keywords:** remote sensing, data-driven breeding, phenomic selection, multi-omic integration, environmental covariates, harvest index, fruiting efficiency, vegetation indices

## Abstract

Optimizing biomass partitioning is essential for achieving sustainable yield improvement in wheat, particularly under increasing environmental stress. Traits such as spike partitioning index (SPI), harvest index (HI), and fruiting efficiency (FE) are central to understanding how assimilates are allocated between vegetative and reproductive organs. However, their complex physiology and the difficulty of manual phenotyping have limited their routine use in breeding programs. This study assessed the potential of unmanned aerial vehicle (UAV)-based hyperspectral reflectance data to predict biomass partitioning traits and related yield components in wheat. Three trials of facultative soft wheat lines (2022–2024) and an independent validation set of advanced breeding lines were used to develop genomic prediction (GP), phenomic prediction (PP), and integrated multi-omic models combining genomic, phenomic, and environmental covariates (ECs). Kernel-based best linear unbiased prediction (BLUP), and machine-learning based, random forest regression and partial least squares regression were implemented to estimate predictive ability (PA). Phenomics-driven models markedly outperformed GP across most traits, achieving PA up to 0.61 for SPI, 0.56 for FE, 0.71 for grains/m^2^ (GN), and 0.66 for grain yield (GY). Hyperspectral data provided higher accuracy than vegetation indices, and multi-omic integration slightly improved prediction (PA up to 0.73 for GN). These results demonstrate that UAV-based hyperspectral phenotyping can effectively capture canopy-level physiological signals associated with biomass partitioning, offering a scalable and data-driven approach for in-season selections. This can help wheat breeding programs to optimize biomass partitioning in modern wheat cultivars for long-term yield resilience and genetic gain.

## 1 Introduction

Biomass partitioning traits in wheat (*Triticum aestivum* L.) determine how the plant allocates resources for structural support, photosynthesis, and grain development. Key traits include the spike partitioning index (SPI), which measures the proportion of biomass allocated to spike at anthesis +7 days (A + 7), fruiting efficiency (FE), which reflects how effectively spike biomass is converted into grain set, and harvest index (HI), grains-to-total-biomass-ratio, a critical indicator of overall resource-use efficiency (Rivera-Amado et al., 2019; Shahi et al., 2025, 2025). Optimizing these traits is essential for improving grain number (GN) and grain yield (GY). Despite a theoretical HI limit of ~65%, current values remain around 50% in spring wheat and 50–55% in winter wheat, suggesting a substantial yield gain if this gap is narrowed (Aisawi et al., 2015). However, traditional destructive sampling methods require multiple labor-intensive processes, including sample collections, drying, separation of plant parts, and dry weight measurements. These challenges limit large-scale assessment in breeding programs (Rivera-Amado et al., 2019; Sierra-Gonzalez et al., 2021; Kunwar et al., 2025), thereby restricting ample genetic exploration and improvement of these complex traits, ultimately slowing progress towards sustainable yield gains. Overcoming this requires high-throughput, non-destructive phenotyping tools that enable rapid trait assessment and resource efficient selections from thousands of germplasm pools.

The advent of high-throughput genomic sequencing revolutionized breeding programs, making genomic selection (GS) one of the most widely adopted techniques in both plant and animal breeding. The GS leverages genome-wide markers and phenotypic data from a training population to predict the breeding values of a testing population that has only been genotyped (Heffner et al., 2011). This approach enables breeders to estimate the genomic breeding values of large germplasm sets without the need for extensive field trials. As a result, many breeding programs have implemented GS to enhance genetic gain and accelerate cultivar development by increasing selection intensity and reducing the breeding cycle (Maulana et al., 2019; Das et al., 2020). However, early GS models did not account for genotype-by-environment (G×E) interactions, limiting their effectiveness in predicting genotype performance across multiple environments.

In recent years, high throughput phenotyping (HTP) has emerged as a powerful tool in plant breeding, enabling large-scale, non-destructive trait assessment that significantly enhances efficiency and accuracy while reducing data collection time. Aerial HTP platforms, in particular, provide a major advantage in multi-environment trials due to their ability to capture high-resolution data rapidly and consistently. These platforms utilize spectral reflectance properties, which vary based on plant structure, pigments, and physiological status, to extract valuable insights into key agronomic traits (Babar et al., 2006; Zheng et al., 2018; Costa et al., 2022). Among HTP-derived features, vegetation indices (VIs), such as the Normalized Difference Vegetation Index (NDVI), have been widely used for assessing plant health and predicting yield-related traits in wheat (Crain et al., 2018; Guo et al., 2020). However, traditional VIs rely on a limited number of spectral bands, which may not fully capture the complexity of plant responses (Montesinos-López et al., 2017). Hyperspectral imaging addresses this limitation by collecting detailed reflectance information across a broad range of wavelengths, improving trait estimation accuracy.

Beyond individual trait prediction, HTP data play a crucial role in improving GS models by indirectly capturing G×E interactions (Rutkoski et al., 2016; Crain et al., 2018; Krause et al., 2019; Robert et al., 2022; McBreen et al., 2025a). Further, recent advancements have extended the genomic best linear unbiased predictor (GBLUP) model to account for G×E interactions (Jarquín et al., 2014; Lopez-Cruz et al., 2015). The availability of environmental covariates (ECs) from weather stations has made it easier to characterize environments, further refining the prediction accuracy (PA) of complex traits across environments compared to single-environment models (Monteverde et al., 2019; Canella Vieira et al., 2022). Integrating multi-omics data, including HTP, ECs, and genomic markers, provides a comprehensive framework to account for trait physiology and G×E interactions for complex traits.

Leveraging multi-omics data in predictive models can be challenging due to the high dimensionality of predictors, which can lead to computational complexity. The Genomic BLUP (GBLUP) model addresses this challenge through an approach that is mathematically equivalent to ridge regression but uses a genomic relationship matrix to efficiently account for genome-wide markers when their number exceeds the number of observations. Similarly, high-dimensional hyperspectral imaging data can be integrated using similar techniques (Krause et al., 2019). ECs can also be incorporated by using a covariance structure (Jarquín et al., 2014). While these statistical approaches improve computational efficiency in multi-omics prediction, machine learning (ML) methods can be further benefited by enhancing the learning process from diverse data types (Gong et al., 2019). For example, convolutional neural networks (CNNs) integrated genomic, environmental, and management data to predict grain yield (Washburn et al., 2021), while tree-based ensemble models, such as random forests (Breiman, 2001) and gradient boosting (Fernandes et al., 2024), improve robustness by incorporating G×E interactions.

Genomic prediction has been previously explored for biomass partitioning traits in wheat (Shahi et al., 2022; Kunwar et al., 2025); however, the complex nature of traits necessitates further investigations using multiple omics data sources and robust validation strategies to enhance the prediction and selection efficiency. In this study, we utilized aerial HTP with hyperspectral reflectance data to develop phenomic prediction models and integrated multi-omic prediction models to enable in-season selections of wheat genotypes for optimized biomass partitioning. This is among the first studies to explore the use of aerial HTP-derived data for biomass partitioning traits prediction in wheat. The main objectives of this study were to: 1) develop phenomic prediction tools for complex biomass partitioning traits in wheat using hyperspectral reflectance data and compare the prediction performance with genomic prediction models; 2) propose multiomic prediction models by integrating genomic markers, hyperspectral reflectance data, VIs, ECs and their interactions, deploying BLUP and ML approaches; and 3) validate the performance of the prediction models using a separate test trial with advanced wheat breeding lines.

## 2 Materials and methods

### 2.1 Plant materials and phenotyping

Field trials were conducted from 2022 to 2024 at the Plant Science Research and Education Unit (PSREU) in Citra, Florida, USA (29.40556°N, −82.16945°W) for phenotyping. The study included 341 elite soft wheat lines developed by the SunGrains™ cooperative breeding initiative, which includes seven public university wheat and small grains breeding programs in the southern United States. Wheat lines were evaluated over three consecutive growing seasons, with 156, 297, and 290 lines tested in 2022, 2023, and 2024, respectively. Of these, 112 lines from the 2022 trial were retained for evaluation in subsequent years, while 290 lines were common between 2023 and 2024. In 2024, a separate validation trial was established at the same experimental site, comprising 120 advanced breeding lines developed by the University of Florida’s small grain breeding program. The breeding lines in validation set did not overlap with the training population but shared some common pedigree relationships and were used to evaluate model performance under realistic breeding conditions. These lines are facultative, with low vernalization requirements, and therefore well suited to the diverse environmental conditions of the southern United States region. More details on the plant materials are provided in Kunwar et al. (2025). The weather patterns during the growing seasons of all three years are summarized in **Supplementary Table S1**. These weather variables were also used to compute the environmental covariance structure (W) for prediction models, which captures similarity among environments based on shared meteorological conditions. Details on the computation of this covariance structure are provided in Section 2.5.3.

Field trials were arranged in an incomplete block alpha-lattice design with two replications per genotype. Seeds were sown in 7-row plots, each plot measuring 3.70 m × 1.77 m, at a uniform seed rate of 100 kg ha^−1^. The PSREU team assisted with planting, trial management (including irrigation, fertilization, and chemical treatments), and harvesting.

Genotypes were evaluated for phenology and five complex traits: spike partitioning index (SPI), harvest index (HI), fruiting efficiency (FE), grain number (GN), and grain yield (GY). Days to anthesis (DTA) was recorded as the number of days from planting to when at least 50% of plants in a plot flowered (Zadoks et al., 1974). SPI was measured at anthesis +7 days (Zadoks scale: GS70) by harvesting all above-ground plant material from a 0.25 m² area within each plot. The samples were oven-dried at 60°C for 72 hours, and SPI was calculated as the ratio of spike dry weight to total above-ground biomass (Shahi et al., 2025).

At physiological maturity (Zadoks scale: GS90), a second set of plant samples was collected from the same plots and oven-dried. The total above-ground biomass was measured, and the samples were threshed to determine grain weight. The HI was calculated as the ratio of grain weight to total above-ground biomass of the sample. GY (g m^−2^) was measured by harvesting all plants in each plot using a combine harvester and adjusting the yield to 13% moisture content. TGW was recorded by counting and weighing a sample of 1000 grains, and GN was determined by dividing GY (g m^−2^) by the average grain weight. FE was calculated as the ratio of GN at maturity to spike dry weight measured at anthesis +7 days (Shahi et al., 2025).

### 2.2 Genotyping

All the methods for the genotyping process are described in detail in our previous studies (Kunwar et al., 2025; McBreen et al., 2025a). In brief, high-quality DNA was extracted from leaves of 2-week-old plants using the sbeadex plant maxi kit on an oKtopure automated extraction system and genotyping-by-sequencing (GBS) library was constructed using two restriction enzymes, *Msp*I and *Pst*I-HF (Poland et al., 2012). Single nucleotide polymorphisms (SNPs) were called using the TASSEL 5GBSv2 pipeline version 5.2.35 (Glaubitz et al., 2014). To ensure data quality, we filtered raw molecular marker data by removing markers with more than 80% missing values, a minor allele frequency below 0.05, or heterozygosity greater than 10%. After filtering, 34,238 SNP markers were retained from the initial 37,395. The missing data were then imputed using Beagle software (Browning and Browning, 2016) for further analysis. The population structure was analyzed through discriminant analysis of principal components (DAPC) implemented in the R package ‘adegenet’ (Jombart et al., 2010). The optimal number of genetic clusters was determined based on the Bayesian information criterion (BIC) calculated within the DAPC framework.

### 2.3 Spectral data acquisition

Spectral data were collected using two unmanned aerial vehicles (UAVs), each equipped with a different imaging system. The first UAV, a quadcopter (Matrice 300, DJI, Shenzhen, China), carried a multispectral imaging system (MicaSense Altum PT, AgEagle, 2024). The second UAV, a hexacopter (Matrice 600 Pro, DJI, Shenzhen, China), was equipped with a hyperspectral sensor (Pika L 2.4, Resonon, Bozeman, MT, USA), which had a 17 mm focal length lens, a 17.6°field of view (FOV), and an instantaneous field of view (IFOV) of 0.71 mrad. The UAV with the multispectral camera conducted two flights: one just before manual phenotyping at the GS70 stage and another two weeks later, while the UAV with the hyperspectral sensor was flown once at the GS70 stage. All flight missions were controlled using the Pix4D Capture application (Pix4D S.A., Prilly, Switzerland) on an iPad (Apple, Cupertino, CA, USA).

To ensure accurate calibration, the multispectral camera was calibrated before each flight using a MicaSense reflectance panel, and four ground control points (GCPs) were placed in the field for precise post-processing. For hyperspectral imaging, a light grey calibration tarp was positioned within the data collection area to facilitate accurate reflectance calibration. Flights were conducted at an altitude of 30 m above ground level with a speed of approximately 1.5 m/s. The image capture settings included an 85% front overlap and a 70% side overlap.

Raw multispectral images were processed using Pix4Dmapper software to generate orthomosaics of the entire field for five spectral bands: Blue (455–495 nm), Green (545–575 nm), Red (650–685 nm), Red edge (705–725 nm), and Near-infrared (820–860 nm). The orthomosaics were further processed in QGIS, where soil masking was applied using an iterative threshold determined from the images to remove non-vegetative pixels. After soil masking, plot-level grids were created and manually adjusted to ensure the ROIs were fully contained within each plot. The average reflectance value for each plot was calculated using the Zonal Statistics plugin in QGIS. Two vegetation indices (VIs), normalized difference vegetation index (NDVI) and enhanced vegetation index (EVI), were derived based on their previous applications in predicting agronomic traits in wheat and other crops. The details of these VI calculations are provided below in **Table 1**.

**Table 1:** Details of vegetation indices used in this study. VIs, vegetative indices; NDVI, normalized difference vegetation index; EVI, enhanced vegetation index.

VIs	Formula	Descriptions
NDVI (Tucker, 1979)	$\frac{\left(NIR-Red\right)}{\left(NIR+Red\right)}$	NIR= Reflectance value in 820–1000 nm waveband Red= Reflectance value in 663–673 nm waveband
EVI (Huete et al., 1997)	$\frac{\left(NIR-Red\right)}{\left(NIR+6\;Red-7.5\;Blue+1\right)}\times 2.5$	NIR and Red denotes same as above Blue = Reflectance value in 465–485 nm waveband

**Figure 1:**
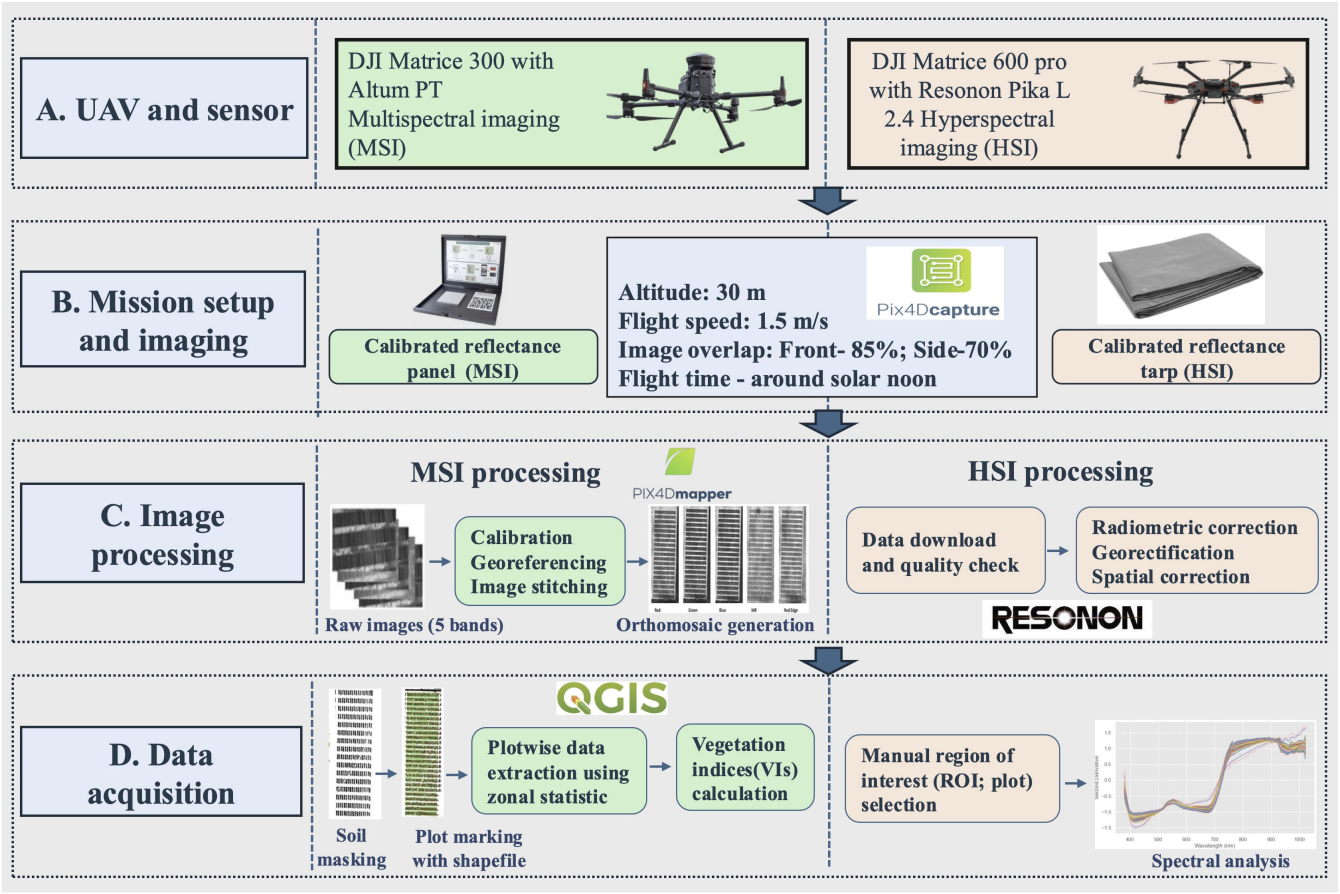
Workflow diagram illustrating the steps for UAV-based hyperspectral (HSI) and multispectral imaging (MSI) data collection and processing. Steps shared by both imaging techniques are highlighted in light blue, MSI-specific steps in light green, and HSI-specific steps in light orange. UAV, uncrewed aerial vehicle.

The high-dimensional spectral data obtained from the hyperspectral sensor were calibrated and analyzed using Spectronon software (Spectronon Pro, Resonon, Bozeman, MT, USA). Georectification and radiometric corrections were applied to integrate GPS/IMU data and ensure accurate radiometric calibration. Regions of interest, defined as the plot areas manually delineated within each UAV image, were selected to extract the average reflectance value for each plot. The hyperspectral system captured reflectance data across 273 spectral bands, covering a wavelength range from 398.47 nm to 1000.58 nm. The details of UAV image collections, calibration, processing, and data acquisition are presented in **Figure 1**. To reduce noise and ensure comparability of spectra, the raw reflectance data were normalized using L2 normalization.

### 2.4 Statistical analysis

Phenotypic, spectral reflectance data, and VIs were analyzed using a linear mixed model using the “lme4” and “lmerTest” packages in R (Bates et al., 2015), focusing on three key analyses: (1) analysis of variance (ANOVA), (2) calculation of broad-sense heritability, and (3) estimation of adjusted means for each trait across genotypes. In this study, the term “environment” refers to the specific growing conditions experienced during a given year. The ANOVA was conducted using model below (Equation 1), which accounted for environmental effects and genotype-by-environment (G×E) interactions by combining data from all years.

(1) $$y_{ijklm}=\mu +g_{i}+E_{j}+\;{\left(gE\right)}_{ij}+B_{k\left(j\right)}+R_{l\left(jk\right)}+C_{m\left(jk\right)}+e_{ijklm}$$

where $y_{ijklm}\;$ represents the observed phenotypic trait value, or hyperspectral reflectance value, or VI; μ is the overall mean; $g_{i}$ is the fixed effect of the $i^{th}$ genotype ( i = 1 to 341); $E_{j}$denotes the fixed effect of the $j^{th}$ environment ( j = 1 to 3); ${\left(gE\right)}_{ij}\;$ accounts for the fixed interaction between the $i^{th}$ genotype and the $j^{th}$ environment. The term $B_{k\left(j\right)}$ represents the random effect of the $k^{th}$ incomplete-block ( k = 1 to 2), $R_{l\left(jk\right)}$ and $C_{m\left(jk\right)}$ captures the random effect of the $l^{th}$ row ( l = 1 to n) and $m^{th}$ column ( m = 1 to m), respectively, both nested within the $k^{th}$ incomplete-block of the $j^{th}$ environment. The term $e_{ijklm}$ represents the residual accounting for variability not explained by other model terms. The *H^2^* was calculated by treating all factors of model (1) as a random effect, using the following formula (Equation 2):

(2) $$H^{2}=\frac{\sigma_{G}^{2}}{\left(\sigma_{G}^{2}\;+\;\frac{\sigma_{G\times E}^{2}}{n}\;+\;\frac{\sigma_{e}^{2}}{nr}\right)}$$

where $\sigma_{G}^{2}$, $\sigma_{G\times E}^{2}$ and $\sigma_{e}^{2},$ indicates variance components due to genetic, genotype-by-environment interactions, and residual effects, respectively, and n and r represents the numbers of environments and replications, respectively.

The adjusted means for all phenotypic traits, spectral reflectance data, and VIs were calculated using the best linear unbiased estimates (BLUEs). These estimates were computed separately for each environment using model below (Equation 3). The model used for these calculations is detailed below:

(3) $$y_{ijkl}=\mu +g_{i}+B_{j}+R_{k\left(j\right)}+C_{l\left(j\right)}+e_{ijkl}$$

All terms here represent exactly the same as defined in model (1), with the analysis conducted separately for each environment. To account for confounding effects in the adjusted means of traits due to differences in the maturity of different genotypes, days to anthesis (DTA) was included as a covariate in the model. Pearson’s correlation among traits was calculated from BLUEs from each environment separately.

### 2.5 Prediction modeling approaches

Prediction models were developed using two main approaches. The first approach utilized the GBLUP model implemented in the BGLR package (Pérez and De Los Campos, 2014). This method allows for both single-kernel models and multi-kernel models that integrate diverse data types using Reproducing Kernel Hilbert Space (RKHS). The second approach employed machine learning (ML) models, specifically Random Forest Regression (RFR) and Partial Least Squares Regression (PLSR). These models were implemented using the Scikit-learn library in Python. The details of each model are provided below:

#### 2.5.1 Genomic effects model (G)

The adjusted means of phenotypic values from all environments jointly analyzed using a genomic main effect model were fitted as below (Equation 4):

(4) $$y_{ij}=\mu +g_{i}+E_{j}+e_{ij}$$

where $y_{ij}$ represents the adjusted means of phenotypic values for ${\text{i}}^{th}$ genotype and $j^{th}$ environment, μ is the overall mean, $g_{i}$ is the genomic main effect of markers corresponding to ${\text{i}}^{th}$ genotype, $E_{j\;}$ is the effect of the environment (year), and $e_{ij}$ represents the residual effects. The vectors g and e are assumed to follow a multivariate normal distribution, which can be written as $g=\left\{g_{i}\right\}\sim N\left(0,G\sigma_{g}^{2}\right)$ and $e\sim N\left(0,I\sigma_{e}^{2}\right)$, where G and I are the genomic relationship matrix and identity matrix, respectively. The matrix *G* was computed by using 34,238 SNP markers data in R statistical software by using “AGHmatrix” package (Amadeu et al., 2023) and the genomic relationship matrix ( G) was expanded to match the phenotypic data from multiple environments by using $\left(ZG\right)Z^{T}$ where Z is the design matrix made with genotypic ID column repeated in multiple environments. $\sigma_{g}^{2}$ and $\sigma_{e}^{2}$ represent the additive genetic and residual variance, respectively.

#### 2.5.2 Hyperspectral effects model (H)

To simulate a scenario where genomic marker is unavailable, phenomic prediction model was developed using similarity matrices derived from hyperspectral reflectance data instead of SNP markers. The adjusted means of hyperspectral reflectance data from all environments were combined before computing the similarity matrix. The phenomic model was fitted as below (Equation 5):

(5) $$y_{ij}=\mu +h_{ij}+E_{j}+e_{ij}$$

#### 2.5.3 Multi-omic Model (G+H+V+W+GW)

This model integrates genomic effects (G), hyperspectral effects (H), vegetation indices (VIs) effects (V), ECs (W) and genotype-by-ECs interactions (GW). The multi-kernel model was fitted as below (Equation 6):

(6) $$y_{ij}=\mu +g_{i}+h_{ij}+v_{ij}+w_{j}+gw_{ij}+e_{ij}$$

where $y_{ij}$, μ, $g_{i}$, $h_{ij}\;$ and $e_{ij}$ are as defined in model (4 and 5). The term $v_{ij}$ represents the main effect of the VIs data from ${\text{i}}^{th}$ genotype and $j^{th}$ environment, with $v=\left\{v_{ij}\right\}\sim N\left(0,V\sigma_{v}^{2}\right)$, where V is the covariance matrix (861× 861) based on vegetative indices and $\sigma_{v}^{2}$ represents the corresponding variance component. *V* was computed as $\frac{SS^{\prime }}{nindices}$, where S is a matrix (861× 4) of centered and standardized adjusted means of vegetative indices collected at both time points and nindices is the total number of vegetative indices ( nindices=4).The term $w_{j}$ represents the EC kernel effect for the $j^{th}$ environment and follows a multivariate normal distribution: $w=\left\{w_{j}\right\}\;\sim \;N\left(0,W\sigma_{w}^{2}\right)$, where W  is a covariance matrix based on ECs and $\sigma_{w}^{2}$ represents the corresponding variance component. W was computed as $\frac{MM^{\prime }}{Q}$, where *M* is a matrix of centered (mean-centering) and standardized (z-score standardization) meteorological covariates (ECs), and Q is the total number of ECs used. Eleven different ECs, including temperature, relative humidity, rainfall, solar radiation, wind, and evapotranspiration (ET), were used. The ECs were collected in a week interval throughout the growing season in each trial and the details are provided in **Supplementary Table 1**. The term $gw_{ij}$ represents the interaction between the *i^th^* genotype and the {it}j{sp}th{/it} {/sp}environment. This interaction term vector ( gw) follows a multivariate normal distribution $gw\sim N\left(0,\;Z_{g}GZ_{g}^{'}\;\circ \;Z_{E}WZ_{E}^{'}\sigma_{gw}^{2}\right)$, where $Z_{g}$ and $Z_{E}$ are the incidence matrices that connect phenotypes with genotypes and environments, respectively. The symbol, ∘ is the Hadamard product and $\sigma_{gw}^{2}$ is the corresponding variance component. The details on computing the W matrix based on ECs and on computing genotype-by-ECs interactions (GW) can be found in Jarquín et al. (2014).

#### 2.5.4 Machine learning models

Machine learning models were used to evaluate predictive performance alongside the multi-kernel models. Two machine learning approaches, random forest regression (RFR) and partial least squares regression (PLSR), were developed. Relationship matrices or covariance matrices, as described above, were used as predictors for genomic and phenomic models. For multi-omic integrations, predictors were combined using a left join function using the columns “Year” and “Genotype” to ensure proper alignment of data across different sources. All machine learning analyses were conducted using the Scikit-learn library (Pedregosa et al., 2011) in Python 3.12.

The RFR is an ensemble learning method that relies on multiple decision trees and is widely applied for predicting complex agricultural traits (Sandhu et al., 2021; McBreen et al., 2025b). In this study, the number of trees was set to 100 (n_estimators=100) to balance computational efficiency and predictive accuracy. The number of features considered at each split (max_features) was set to the default, which uses all available predictors for regression. The default learning rate of 0.1 was used, and a random seed (random_state=42) was specified to ensure reproducibility.

The PLSR was also implemented, as it is well-suited for handling multicollinearity in datasets with a large number of predictors. The model was fitted using the “cross_decomposition” function from Scikit-learn (Pedregosa et al., 2011), with a maximum number of iterations set to 200 (max_iter=200) to ensure convergence. The optimal number of latent components was selected through cross-validation within the training dataset by minimizing the mean squared error, ensuring good predictive performance while avoiding overfitting.

### 2.6 Cross validations and evaluation metrics

**Figure 2:**
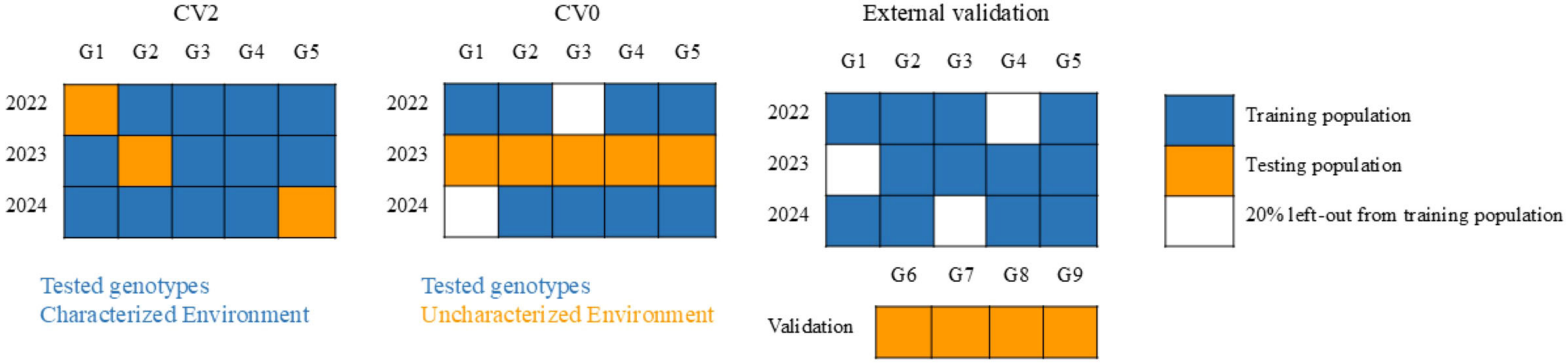
Schematic illustration of different cross-validation schemes adopted in this study with 5 genotypes in multi-year trials and 4 genotypes in the breeding validation trial.

Three types of cross-validation schemes were used to evaluate the robustness of the prediction models (**Figure 2**). All prediction models were initially evaluated using a CV2 cross-validation scheme, where the phenotypic data was randomly divided into five subsets (K = 5). The models were trained on four subsets (80% of the total data) and tested on the remaining fifth subset (20%). All BLUP models were run for 12,000 iterations with a 2,000-iteration burn-in, and a fixed random seed [set.seed (1)] was set to ensure reproducibility. The ML models were evaluated using the same five-fold cross-validation strategy with shuffled splits at the genotype level, and a fixed random seed (random_state = 42) was used for reproducible data partitioning and model training. Predictive ability (PA) was assessed by calculating Pearson’s correlation coefficient between the predicted values and the observed values by combining the datapoints from all three environments.

The second validation scheme (CV0) tested the ability of the models to predict in a new environment. In this scheme, training data from two years were combined, and the model was tested in a third year that was left out entirely. For robust evaluation, the combined training dataset was further divided into five folds; in each iteration, four folds (80%) were used to train the model, and predictions were made for the left-out environment. This process was repeated five times, rotating the folds within the training data. Pearson’s correlation coefficient was again used to quantify predictive ability.

To assess the models’ capacity to predict the performance of new genotypes, additional validation was performed using a separate breeding trial with advanced wheat lines. Similar to the previous validation methods, the entire training dataset (from all environments) was divided into five folds, and models were trained on four folds at a time to predict external validation breeding lines. The details of different validation schemes are presented in **Figure 2**. Pearson’s correlation coefficient was used to evaluate model performance. Additionally, to assess the models’ reliability in selecting superior genotypes in breeding trials, the top 30 breeding lines were identified using the predicted breeding values from the models and compared with rankings based on the adjusted means of manually phenotyped traits. This comparison was expressed as a coincidence index (CI).

## 3 Results

### 3.1 Descriptive statistics

The study evaluated 341 elite soft wheat lines over three years (2022, 2023, and 2024) and 120 advanced breeding lines for the validation study (2024). The descriptive statistics of both phenotypic traits and VIs for each trial are presented in **Supplementary Table S2**. Both SPI and HI showed considerable genotypic variation while maintaining relatively stable means across environments (SPI: 0.34-0.35; HI: 0.34-0.37). In contrast, FE and GY were more environmentally sensitive, with FE means ranging from 46.6 (2024) to 70.4 (2022) and GY from 406.8 g m^−2^ (2023) and 478.9 g m^−2^ (2022). Similarly, GN mean values varied from 14,100 m^−2^ (validation) to 19,300 m^−2^ (2022). The VIs also revealed clear temporal patterns and environmental responses. The NDVI1 varied from 0.68 to 0.78 and EVI1 from 0.47 to 0.82, both decreasing at later stages (NDVI2: 0.32-0.54; EVI2: 0.17-0.30) as crops matured. Hyperspectral reflectance (**Supplementary Figure S1**) exhibited higher genotypic variation in 2022 and 2023, with greater reflectance in the near-infrared range (700–1000 nm). Broad-sense heritability (H²) estimates (**Supplementary Table S2**) from the combined-environment analysis indicated moderate to high H² for SPI (0.51) and HI (0.77), moderate for FE (0.48) and GY (0.60), and generally higher and more stable H² for VIs (NDVI1: 0.78; NDVI2: 0.76; EVI2: 0.74).

**Figure 3:**
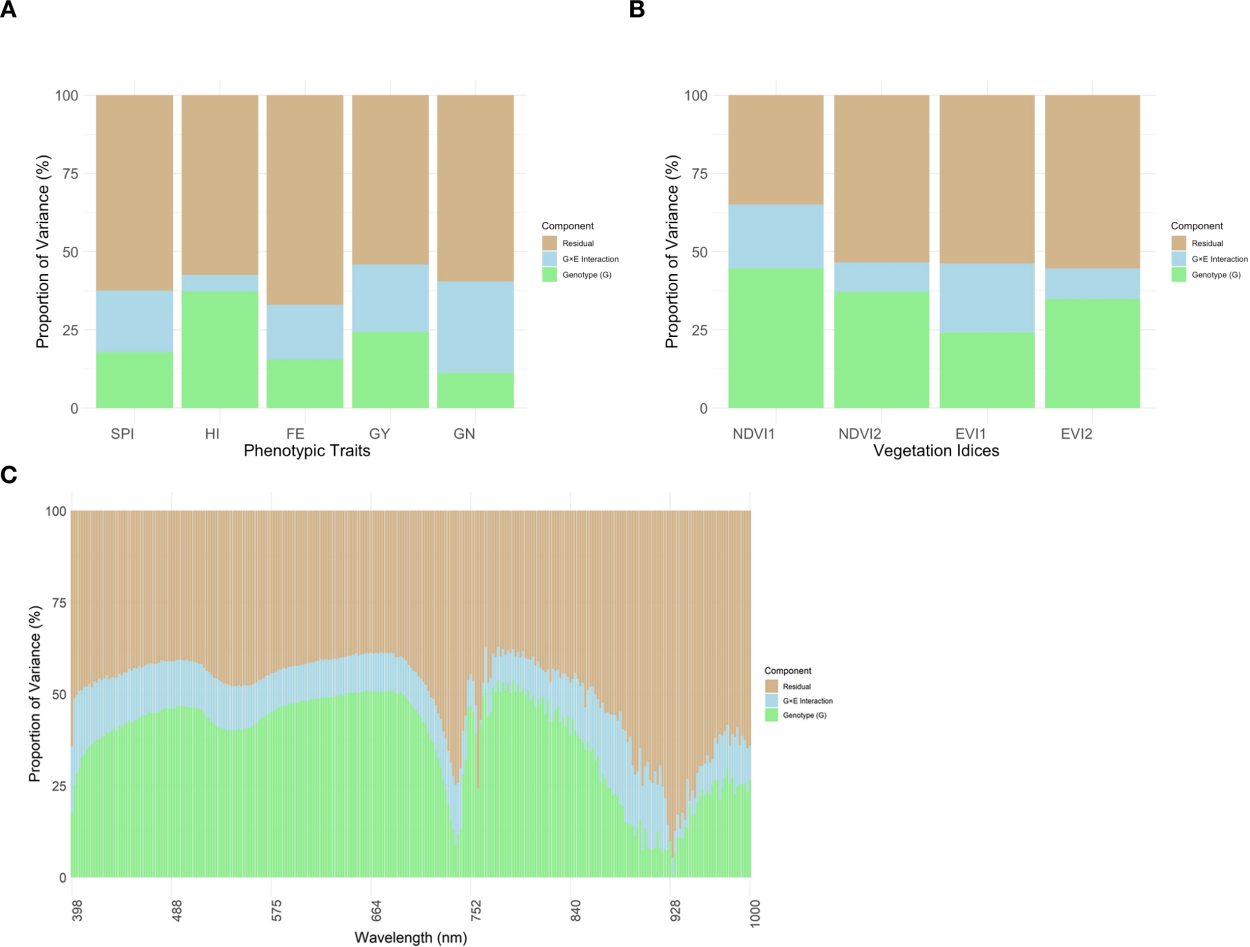
Proportion of variance governed by genetic (G), G×E, and residual for various phenotypic traits **(A)**, VIs **(B)**, and reflectance data from all 273 bands of hyperspectral sensor **(C)**. SPI, spike partitioning index at anthesis+7 days; HI, harvest index; FE, fruiting efficiency; GY, grain yield (g m^-2^); GN, grain numbers m^-2^; NDVI, Normalized Difference Vegetation Index; EVI, Enhanced Vegetation Index.

Variance components from the combined multi-environment analysis (**Figure 3A**) revealed that genotype accounted for less than 20% of phenotypic variance for SPI, approximately 35% for HI, and about 15% for FE. GY had a moderate genotypic contribution (~25%) with considerable G×E interaction (~20%), while GN displayed the lowest genotypic effect (~10%) and the highest G×E component (~30%). In contrast, spectral traits showed stronger genetic control, with genotype explaining approximately 45% of NDVI1 variance (**Figure 3B**). For hyperspectral reflectance (**Figure 3C**), genotypic influence varied by wavelength, generally ranging from 25% to 40%, peaking near 750–820 nm and declining beyond 900 nm.

### 3.2 Correlations

Analysis of correlation coefficients (**Supplementary Table S3**) revealed significant relationships among traits and with VIs in each environment. The SPI was positively correlated with HI (r = 0.07 to 0.28***) and negatively with FE (r = -0.22** to -0.39***). HI showed significant positive correlation with GY in all environments (r = 0.19** to 0.52***) except validation (r = 0.01), and GY and GN were strongly correlated (r = 0.72*** to 0.82***). The VIs were generally negatively correlated with SPI and positively associated with FE, GY, and GN; for example, NDVI1 and EVI1 showed negative correlations with SPI (r = –0.22*** to –0.62***) and positive correlations with FE (r = 0.22*** to 0.54***) and GN (up to r = 0.77***). These trends persisted for the second measurement indices, except in 2022, when NDVI2 correlated negatively with GY and GN. Correlations between VIs and HI were weak and mostly non-significant.

**Figure 4:**
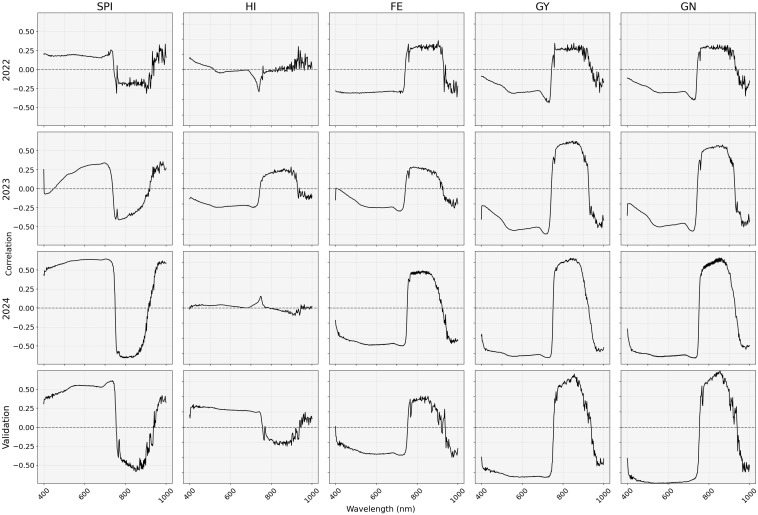
Correlation of phenotypic traits with hyperspectral reflectance data in different wavelength regions in each trial. SPI, spike partitioning index at anthesis+7 days; HI, harvest index; FE, fruiting efficiency; GY, grain yield (g m^-2^); GN, grain numbers m^-2^.

Hyperspectral reflectance data revealed clear wavelength-specific trends (**Figure 4**). The SPI showed positive correlations in the visible range (420–720 nm, peaking ~700 nm) and negative correlations in the near-infrared region (750–900 nm, strongest around 850 nm). In contrast, FE, GY, and GN displayed the inverse patterns, with negative correlations at 400–720 nm range and positive correlations in the NIR region. The HI showed minimal correlation across all wavelengths. The red edge region (680–720 nm) and NIR region (880–900 nm) consistently showed the strongest association with multiple traits, highlighting their potential for developing targeted spectral indices for non-destructive trait estimation.

### 3.3 Predictive ability under CV2 scheme

Five groups were identified in the population structure analysis using a principal component analysis (PCA), with the first two principal components explaining 9.7% of the variance (**Supplementary Figure S2**). The distribution of SNP markers across different sub-genomes and chromosomes is presented in **Supplementary Figure S3**.

**Figure 5:**
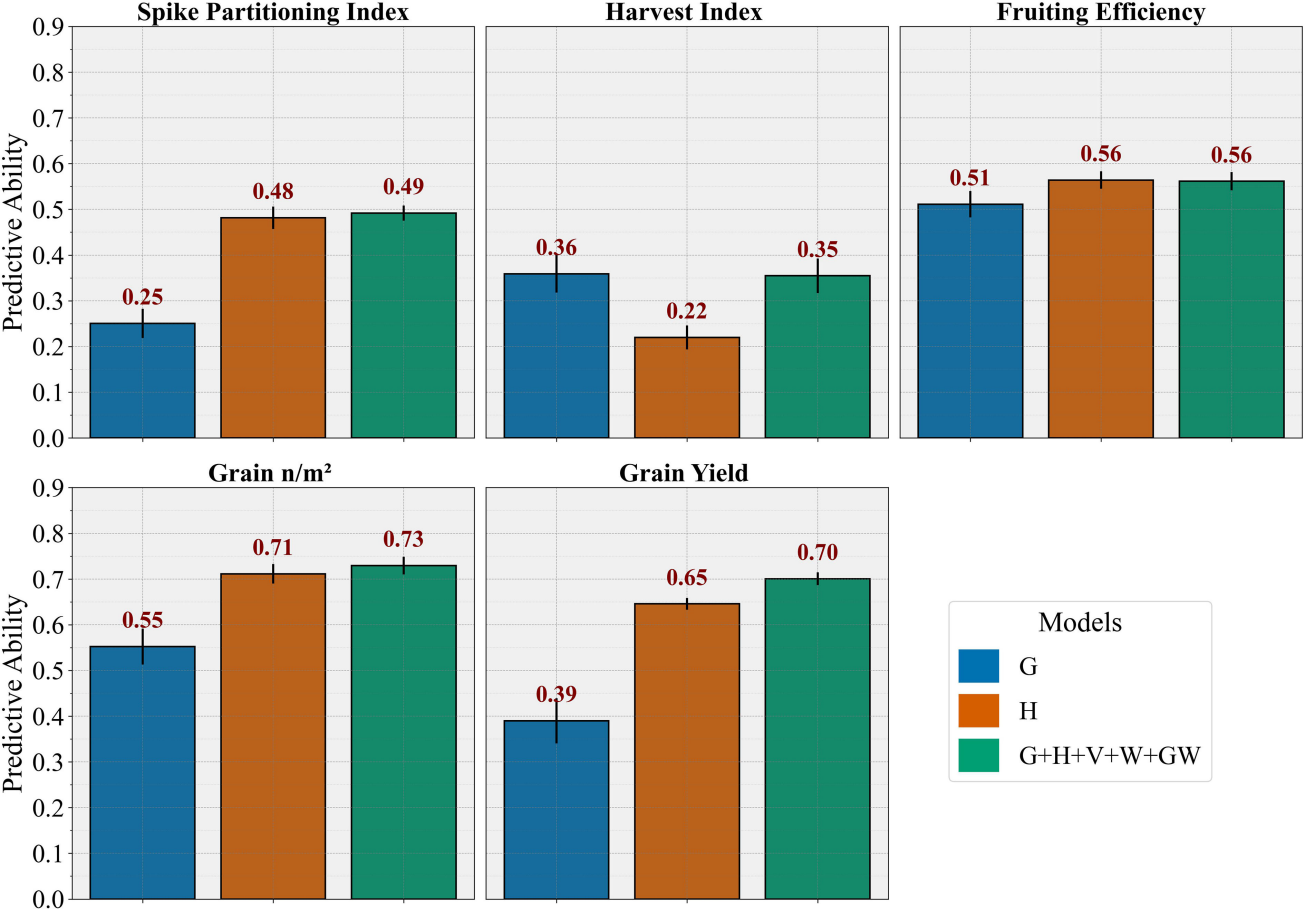
Bar diagrams showing the predictive ability of different kernel-based models (BLUP) to predict phenotypic traits in a 5-fold cross-validation scheme (CV2). G, genomic prediction model; H, hyperspectral-based phenomic prediction model; G+H+V+W+GW, multi-omic model integrating all predictors.

Under the CV2 scheme (**Figure 5**), the genomic prediction model (G) provided moderate PA for most traits, with mean PA ranging from about 0.25 for SPI to 0.55 for GN. Notably, the genomic effect was more informative for HI, where PA reached around 0.36. Models using hyperspectral phenomic data (H) showed higher PA for traits like SPI, GN, and GY, where PA values ranged from 0.22 for HI to 0.71 for GN. For some traits, the improvement in PA from G to H was even 1.5-to 2-fold; for instance, PA for SPI increased from 0.25 (G) to 0.48 (H), and for GY from 0.39 (G) to 0.65 (H). This improvement likely reflects the complex genetic architecture of these traits and greater micro-environmental influence, which the H model captures better than the G model, as the latter uses only additive effects. Combining all data sources in the full multi-omic model (G+H+V+W+GW) produced similar or slightly higher PA for most traits compared to the H model alone. For example, for HI, the multi-omic model achieved a PA of 0.35, improving over the H model’s 0.22; for GY, PA increased modestly from about 0.65 (H) to 0.70 with full integration, highlighting the modest added value of combining genomic, phenomic, vegetation index, and environmental information for predicting complex traits.

Additionally, machine learning models (RFR and PLSR) were tested under the CV2 scheme (**Supplementary Figure S4**). Overall, RFR generally outperformed PLSR across most traits, although both showed lower or comparable predictive ability relative to the main BLUP models. While the H and multi-omic combinations tended to improve ML predictions compared to G alone, the gains were modest.

### 3.4 Leave-one-year-out validation (CV0)

**Figure 6:**
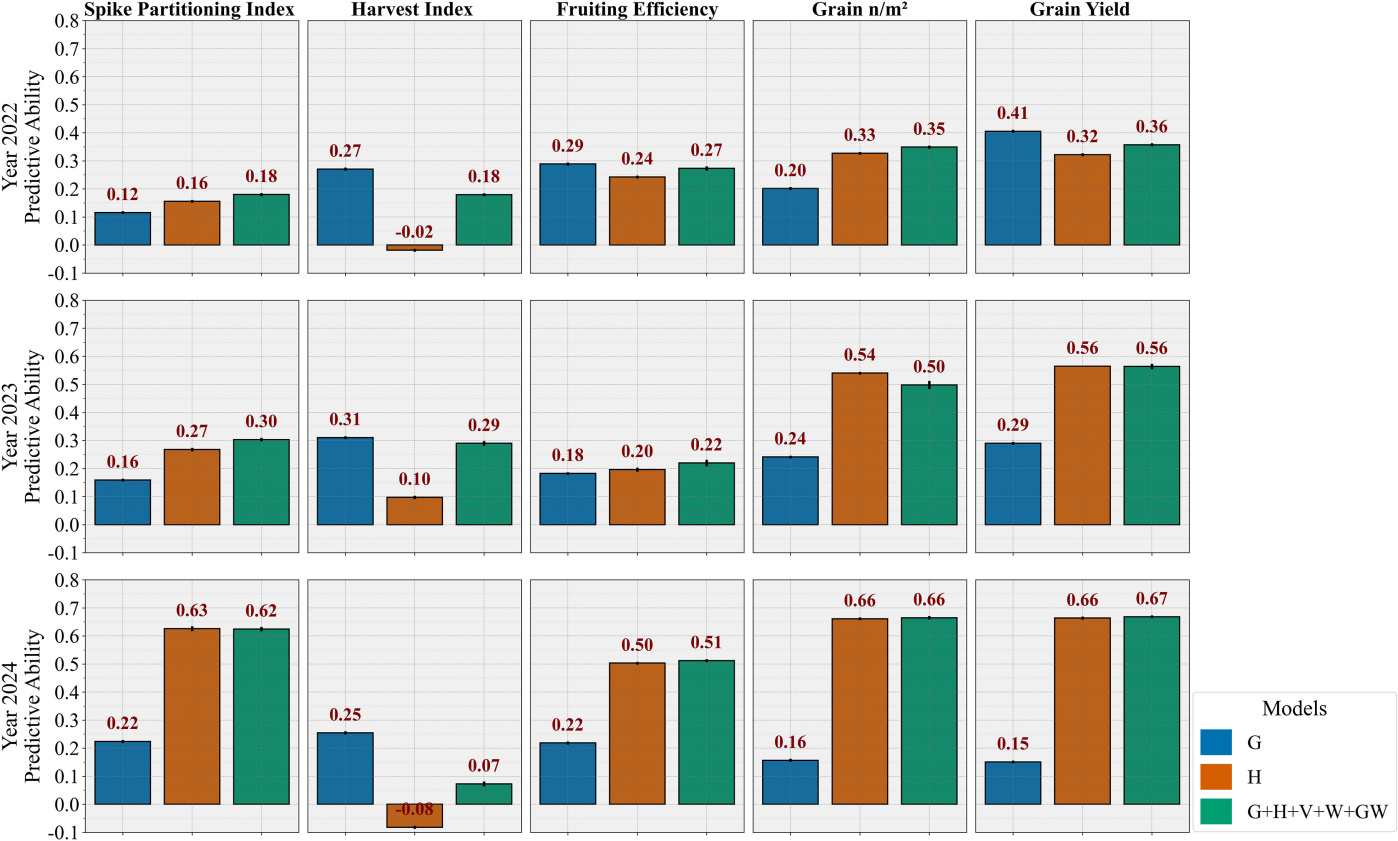
Bar diagrams showing the predictive ability of different kernel-based (BLUP) models to predict phenotypic traits in leave-one-year-out (CV0) cross-validation scheme. Traits for 2022 were predicted using 2023 and 2024 data, and the same was done for the other two years. G, genomic prediction model; H, hyperspectral-based phenomic prediction model; G+H+V+W+GW, multi-omic model integrating all predictors.

The models G, H, and G+H+V+W+GW were further evaluated under the CV0 cross-validation scheme (leave-one-year-out validation), with the BLUP results presented in **Figure 6**. As expected, PA decreased when the test year data were excluded from training.

The G model demonstrated consistent superiority for HI predictions across all three years, with PA ranging from 0.25-0.31, with the highest PA in 2023. For GY, the model showed notable year-specific variation, performing best when predicting year 2022 (0.41) but declining substantially in the following years. For other traits, the model G exhibited low to moderate PA: SPI (0.12-0.22), FE (0.18-0.29), and GN (0.16-0.24), indicating limited cross-year stability for these traits. The H model demonstrated better cross-year generalizability than the G model for all traits except HI. It showed particularly strong and stable performance for GN, with PA ranging from 0.33 to 0.66, and for GY, with PA between 0.32 and 0.66. For SPI and FE, the H model’s PA was highest in 2024 (0.63 and 0.50, respectively) but remained low to moderate in other years. The multi-omic model (G+H+V+W+GW) performed similarly to the H model, with only modest gains for certain traits, such as FE in all years and SPI in 2022 and 2023.

The ML-based approaches (**Supplementary Figure S5**) generally showed patterns similar to the BLUP models, with BLUP maintaining slight superiority in most cases. Notably, the PLSR models performed comparably to or better than the RF models under CV0 conditions, contrasting with CV2 results. The ML models confirmed the overall trend of hyperspectral data superiority for most traits and genomic model consistency for HI predictions across years.

### 3.5 External validation using advanced wheat breeding lines

**Figure 7:**
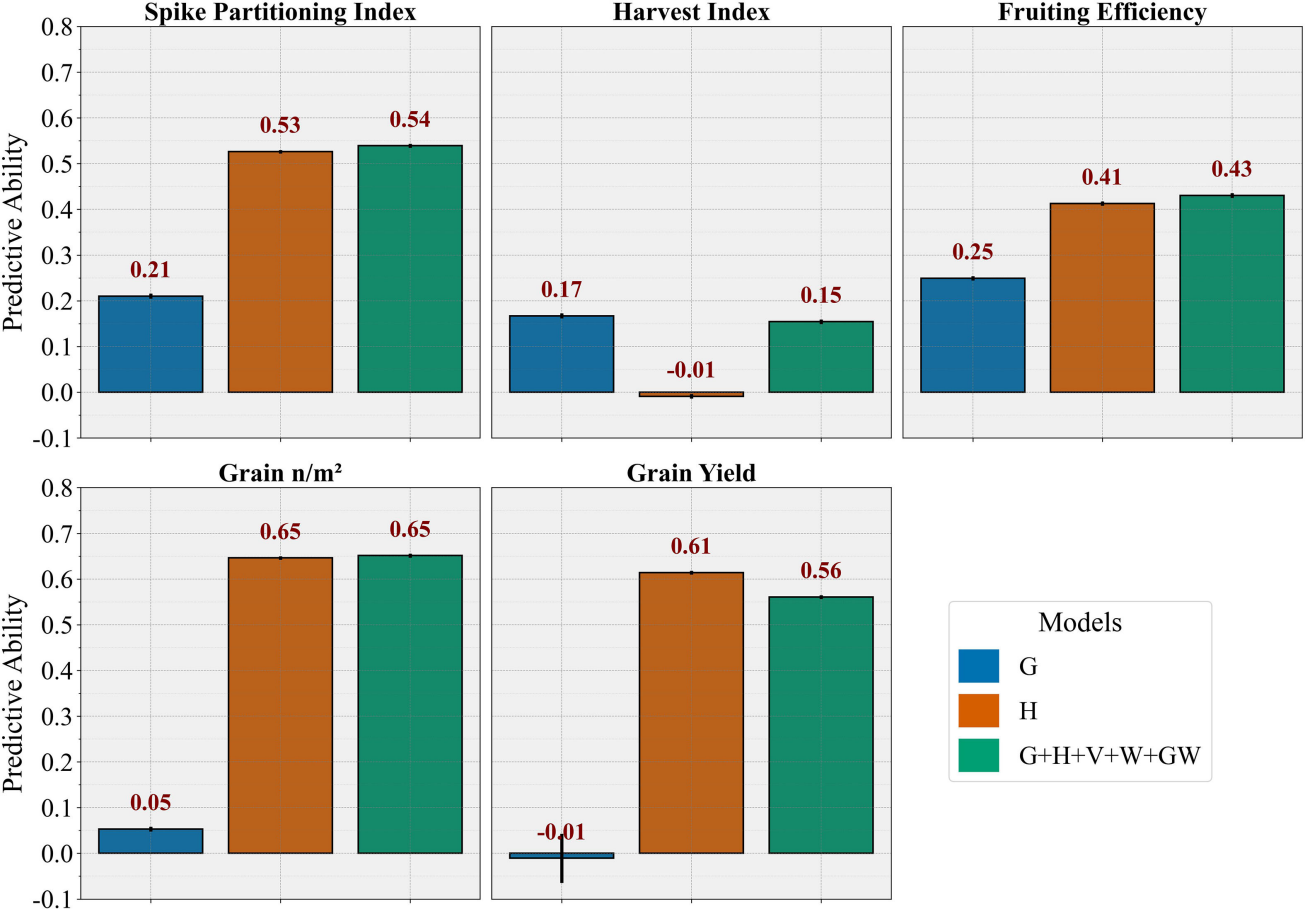
Bar diagrams showing the predictive ability of different kernel-based (BLUP) models to predict phenotypic traits in separate breeding validation trial. The models were used to predict the trait in the whole validation dataset by training the model in 5-folds in the training dataset. G, genomic prediction model; H, hyperspectral-based phenomic prediction model; G+H+V+W+GW, multi-omic model integrating all predictors.

To assess performance under a real breeding scenario, the models were validated using advanced breeding lines (**Figure 7**). Among the BLUP models, the G model demonstrated consistent PA for HI (0.17), superior to other models, maintaining its trait-specific advantage observed in previous cross-validation studies. The G model also achieved moderate PA for SPI (0.21) and FE (0.25) but showed limited effectiveness for yield-related traits when predicting the separate validation trial (GY: -0.01, GN: 0.05). The H model exhibited strong predictive performance across multiple traits, achieving high PA for yield components (GN: 0.65, GY: 0.61) and moderate performance for biomass partitioning traits (SPI: 0.53, FE: 0.41). It showed minimal PA for HI (-0.01), consistent with prior cross-validation results. The multi-omic model (G+H+V+W+GW) again performed similarly to the H model, with minimal or no improvement, consistent with the trends observed in previous cross-validation results.

The ML models tested on external validation using advanced breeding lines (**Supplementary Figure S6**) revealed that ML approaches generally achieved slightly higher performance than the BLUP models, contrasting with the trends observed in the two cross-validation schemes. Both PLSR and RF models confirmed the consistent pattern of genomic data providing optimal HI predictions, while hyperspectral and multi-omics approaches demonstrated superior PA for yield-related and other biomass partitioning traits. Notably, multi-omics integration showed particular strength across most traits in the independent validation scenario, with PLSR models generally outperforming the RF approaches.

### 3.6 Selection of top genotypes in breeding validation trial

**Figure 8:**
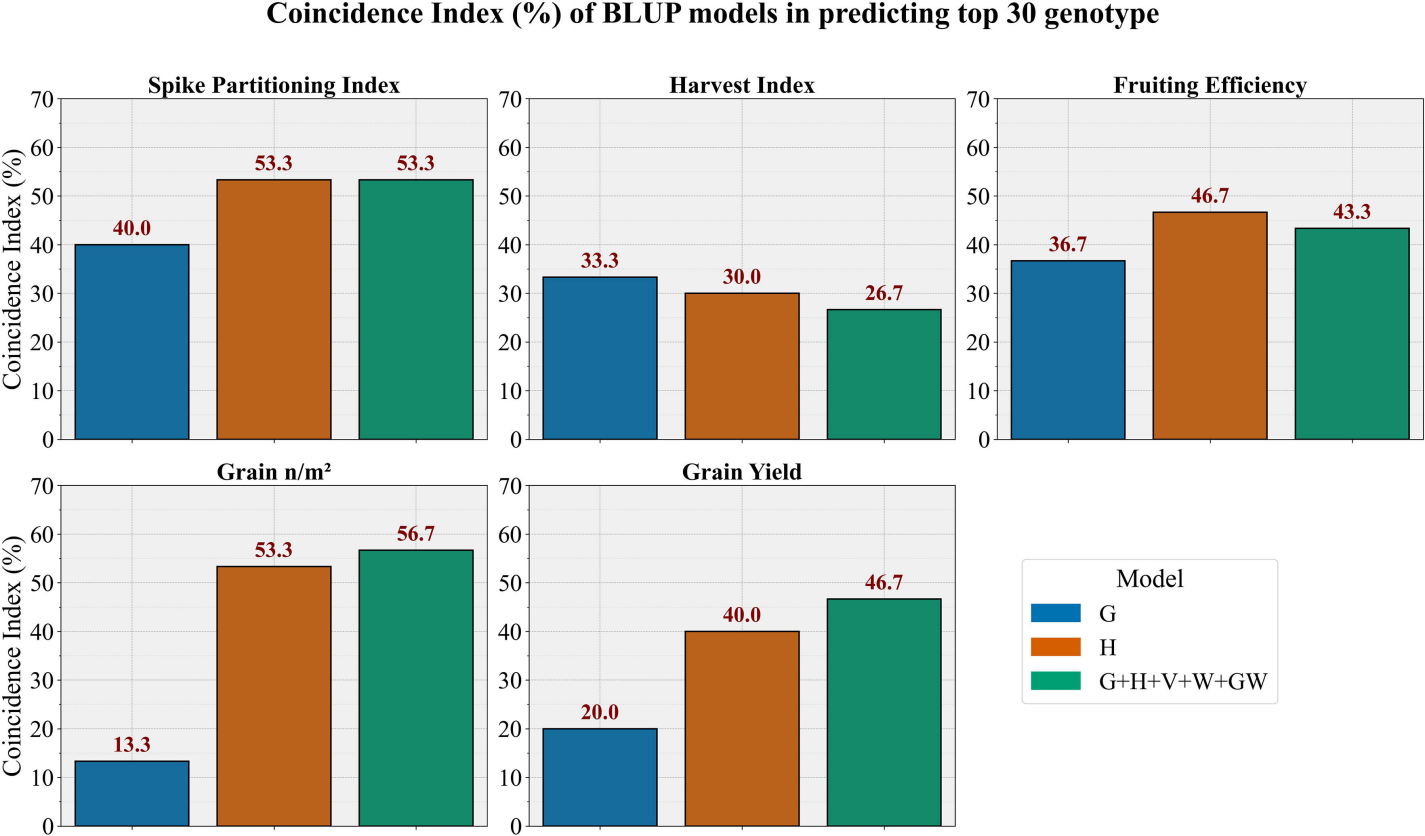
Bar diagrams showing the coincidence index of different kernel-based (BLUP) models in predicting top 30 genotypes vs top 30 genotypes estimated using adjusted means (BLUEs) of manually phenotyped trait. G, genomic prediction model; H, hyperspectral-based phenomic prediction model; G+H+V+W+GW, multi-omic model integrating all predictors.

To further evaluate the effectiveness of the BLUP models for selecting top-performing genotypes, we calculated the percentage similarity between the top 30 genotypes predicted by each model and those ranked by the adjusted means of manually phenotyped traits. This similarity, known as the coincidence index (CI), is presented in **Figure 8**.

The G model demonstrated moderate selection effectiveness with notable trait-specific variation. It achieved reasonable performance for SPI (40.0%) and HI (33.3%), aligning with its predictive strengths, but showed limited utility for selecting yield-related traits, particularly GN (13.3%) and GY (20.0%). The H model exhibited strong genotype selection capability across multiple traits, with high effectiveness for GN (53.3%), SPI (53.3%), and FE (46.7%). It showed moderate performance for GY (40.0%) but reduced effectiveness for HI (30.0%). The multi-omic model (G+H+V+W+GW) displayed the highest selection performance for yield-related traits, achieving the greatest effectiveness for GN (56.7%) and GY (46.7%). It also maintained strong performance for SPI (53.3%) and FE (43.3%) but showed reduced effectiveness for HI (26.7%).

## 4 Discussions

The success of modern plant breeding depends on efficient identification of genotypes with high genetic potential that can deliver stable phenotypic performance across environments. This is particularly critical for complex traits that are costly or impractical to phenotype directly and are significantly affected by G×E interactions. Our study addressed this by exploring how genomic, phenomic, and integrated multi-omic prediction models can help breeders select superior wheat lines for complex biomass partitioning traits. We utilize multiple data types such as aerial high-throughput phenotyping-based hyperspectral reflectance data, VIs, genomic markers, ECs, and marker-EC interactions in predicting traits such as SPI, HI, FE, GN, and GY.

### 4.1 Genetic effects on spectral data and their association with phenotypic traits

Relative to phenotypic traits, the impact of genetic factors on the variance of VIs was more pronounced. The variance decomposition on hyperspectral reflectance data demonstrated wavelength-dependent patterns, where the substantial genotypic variance (25-40%) across most wavelength bands indicates strong genetic control over plant spectral properties, which aligns with findings from Montesinos-López et al. (2017), who reported significant genotypic effects on hyperspectral reflectance in wheat. Similarly, McBreen et al. (2025a) observed consistent moderate to high broad-sense heritability at certain wavelength regions in hyperspectral reflectance data, suggesting significant genetic control.

Phenotypic correlations indicated strong associations between most traits with GY, underscoring their importance in enhancing wheat productivity. Similar association of HI, FE, and GN with GY was also observed in previous studies in wheat (Rivera-Amado et al., 2019; Sierra-Gonzalez et al., 2021; Shahi et al., 2022). However, variations in these associations across different trials suggest that G×E interactions influenced their relationships. A negative correlation between SPI and FE and a positive correlation with HI suggests a potential trade-off in biomass allocation, where an increase in grain size may lead to a reduction in GN. In general, these findings highlight SPI, FE, HI, and GN as key targets for improving GY, though their complex interactions with environmental factors necessitate efficient phenotyping approaches to optimize these traits simultaneously.

Consistent significant correlations of NDVI and EVI with phenotypic traits, except for HI, indicate their usefulness in the prediction of these traits. Spectral reflectance data from HSI revealed wavelength-specific relationships between spectral reflectance and phenotypic traits. GY, GN, and FE showed the strongest negative correlations in the red edge region (680–720 nm). Several studies in remote sensing revealed that the red edge region provides valuable information on leaf area index and biomass quantity, thereby providing insights into general plant health and productivity (Horler et al., 1983; Smith et al., 2004). Similar association of reflectance in the red edge region with GY in wheat has been reported previously in several studies in wheat (Lozada et al., 2019; Shafiee et al., 2024). On the other hand, the strongest positive correlation of these yield-associated traits was observed in the NIR region (880–900 nm). NIR region-based indices and reflectance data have been previously used to predict various agronomic traits and yield in wheat (Babar et al., 2006; Lozada et al., 2019). SPI showed positive correlations in the visible regions and negative correlations in the NIR (750–900 nm). Reflectance in the visible region is primarily influenced by photosynthetic pigments, and in the NIR by canopy structure and mesophyll scattering (Sims and Gamon, 2002; Marín-Ortiz et al., 2020). The comparatively reduced chlorophyll content and altered canopy structure in high spike-to-plant-biomass varieties might have caused these correlation patterns of SPI with reflectance data in our study.

Despite variations in genotypic effects and the strength of correlations with phenotypic traits across different wavelengths, we used reflectance data from all bands to compute the similarity matrix. This approach mirrors the marker-based relationship matrix, which incorporates all SNP markers regardless of their varying effects on the trait. Previous studies have reported that removing low heritable wavelengths didn’t improve the PA for wheat GY (Montesinos-López et al., 2017) and biomass yield in rye (Galán et al., 2020). Similarly, Krause et al. (2019) and McBreen et al. (2025a) also utilized reflectance data from all bands of hyperspectral imaging in predicting wheat yield.

### 4.2 Phenomic and genomic prediction for biomass partitioning traits

Overall, our results highlight that genomic prediction models, which estimate breeding values based solely on additive genetic effects, showed moderate to low PA across all traits, suggesting that genomic information alone inadequately captured the complexity of most traits in our study. In contrast, models using phenomic predictors, such as hyperspectral reflectance, resulted in higher PA for complex traits (except for HI), across both GBLUP and ML approaches. This reflects the ability of phenomic prediction models to capture endophenotypes and G×E interactions in addition to additive genetic information (Rincent et al., 2018; Robert et al., 2022). In our study, moderate to strong correlations between most traits and spectral reflectance or VIs likely contributed to the higher PA values for phenomic models, while HI, which showed weaker correlations with spectral data, also showed lower phenomic PA.

Comparable or higher PA of phenomic prediction models using hyperspectral reflectance than genomic-only models have been previously reported in the prediction of GY in wheat in multiple studies (Krause et al., 2019; McBreen et al., 2025a). Similar observations of phenomic prediction models using NIR spectroscopy in corn (Graciano et al., 2025) in predicting multiple traits and yield in coffee (Adunola et al., 2024) were also observed in recent studies. However, Wang et al. (2025) noted, direct comparison of PA values between genomic and phenomic prediction can be misleading because phenomic models partly capture non-genetic variation, which does not directly contribute to long-term genetic gain. In this study, we addressed this issue by applying a two-step analysis: first, adjusting raw phenotypic data for spatial and external factors to better approximate genetic effects, and then using the adjusted means to compare the performance of prediction models. While this likely reduced some micro-environmental noise, we acknowledge that it may not eliminate all non-genetic factors. Therefore, rather than focusing solely on comparing models based on PA, we also aimed to highlight the unique advantages of each approach across different breeding scenarios and the complementary nature of each omic layer in multi-omic approaches.

Genomic prediction models offer the unique advantage of predicting breeding values before planting, but genomic data can still be costly or unavailable for all breeding materials. Although phenomic approaches using aerial imaging require setting up field trials and collecting in-season data—making purely forward predictions more challenging—they offer scalable, non-destructive methods to screen large numbers of genotypes in the field, which is impractical with traditional destructive phenotyping methods for biomass partitioning traits. Notably, in our study, the genomic model alone showed poor predictive performance in the independent breeding line validation, suggesting that for complex biomass partitioning traits, phenomic information can provide valuable complementary insights when genomic information alone is insufficient.

Phenomic predictions using hyperspectral reflectance data consistently outperformed models based on VIs (data not shown). A similar result was also observed by (Shafiee et al., 2024) on comparing multispectral reflectance data with multiple indices for predicting GY in wheat. However, VI-based prediction is a viable alternative in wheat breeding where hyperspectral imaging is not available, as VI measurements are less complex, cheaper, and computationally less demanding than high-dimensional hyperspectral reflectance data.

### 4.3 Implementing genomic and phenomic prediction in breeding programs

Genomic and phenomic prediction represent two powerful yet distinct approaches for advancing plant breeding programs. Rather than viewing these approaches as direct alternatives, our findings emphasize their complementary roles within an integrated breeding pipeline, as also mentioned in several previous studies (Gonçalves et al., 2021; Robert et al., 2022). The GP provides stable genetic potential from the genome estimated breeding values, which can guide pre-season decisions for parental selection and crossing designs when no field phenotypes are yet available. Once plots are grown, PP models enable rapid, non-destructive in-season screening of large mid-generation populations (F5–F8) to select promising lines based on current crop performance. This offers valuable insights into trait expression and plasticity under specific field conditions. While this can enhance PA for complex traits with strong genotype-by-environment interactions, it also means that PP does not isolate pure genetic merit, as noted by previous studies (Robert et al., 2022; Wang et al., 2025). This two-tiered approach addresses the challenge of manually phenotyping biomass partitioning traits and reduces reliance on destructive sampling. It also mitigates costs when genotyping every line is not feasible.

### 4.4 Challenges associated with the hyperspectral system

A few challenges in using hyperspectral reflectance data for phenomic prediction are the high initial equipment cost and the complexity of handling high-dimensional data (Wang et al., 2024). Since we found promising results using VI-based predictions, the development and use of high-throughput VI-based tools might be a viable, cheaper alternative. By using the similarity matrix-based approach in both kernel-based and ML models, we attempted to reduce the complexity associated with the integration of large-dimensional hyperspectral and genomic data. Maintaining imaging conditions as consistently as possible across years is crucial for ensuring model transferability of hyperspectral-based phenomic predictions, as spectral reflectance data can be influenced by solar irradiance, atmospheric conditions, cloud cover, and other climatic factors. Despite these challenges, with continued advancements in imaging technology and analytical methods, the applicability of hyperspectral-based phenomic prediction is expected to expand, offering a powerful tool to capture GxE interactions, helping in improving complex traits in plant breeding.

### 4.5 Integration of multi-omic data for predicting complex traits

Combining multiple data layers (G+H+V+W+GW) slightly improved PA over single-omic models. This aligns with findings from several recent studies, which similarly demonstrated improved PA with multi-omic models incorporating comparable predictors. However, an important trade-off exists between the added PA and the increased model complexity and computational demand. Also, while our study used the same-season weather data for envirotyping and to capture key G×E interactions, breeders potentially can use historical records, seasonal norms, or forecasts to approximate environmental effects when making predictions for new sites or future seasons (Gillberg et al., 2019). In our study, the improvement by the multi-omic model over the H model was limited, likely because hyperspectral data already captured much of the relevant variation, including endophenotypes and micro-environmental signals. Whether the added complexity and computational cost of multi-omics integration are justified depends on the breeding context. Future research with more diverse environments and additional traits will help clarify when multi-omic integration provides a clear advantage.

### 4.6 Performance of different modeling approaches under different circumstances

Kernel-based approach is one of the widely used methods for integrating multi-omic models (Jarquín et al., 2014; Schrag et al., 2018; Krause et al., 2019; Hu et al., 2021; Ali et al., 2024; Wu et al., 2024; McBreen et al., 2025a). Both RFR and PLSR models have also been widely used to implement in genomic, phenomic, and multi-omic integrations (Shafiee et al., 2024; Graciano et al., 2025). In this study, BLUP models consistently demonstrated superior PA compared to both ML approaches across most traits and data types under CV2. McBreen et al. (2025a) also observed the superiority of multikernel models over different ML-based models when integrating multi-omics data for predicting GY in wheat. However, in the leave-one-year-out validation (CV0), the gap between models narrowed, with PLSR performing comparably to or better than RFR for several traits, suggesting its robustness across growing seasons, possibly due to capturing the most relevant latent variables, minimizing the impact of trial-specific noise. Interestingly, in external validation using advanced breeding lines, ML models, particularly PLSR for complex traits like grain number and grain yield, outperformed BLUP models, while RFR excelled in multi-omic predictions of SPI and FE. This highlights that the optimal modeling approach may vary depending on the prediction context, with ML models potentially offering advantages when applied to genetically distinct material.

### 4.7 Breeding data validation and selection of top-performing genotypes

Our findings indicate that the phenomic and multi-omic models achieved higher PA in the breeding validation datasets and in CV0 predictions for 2024 compared to earlier CV0 predictions (2022 and 2023). This improvement is likely due to the stronger correlations between key traits and spectral reflectance in the 2024 and validation trials. Additionally, while the CV0 predictions used only two years of training data, the breeding validation dataset benefited from a larger training set spanning three years, supporting better generalization. These results align with findings from McBreen et al. (2025a), who reported that increasing the training population size improves PA in forward prediction scenarios.

Importantly, our results highlight the practical value of phenomic and multi-omic predictions for in-season selection. While genomic models, which capture only additive genetic effects, achieved moderate CI (~35%) for traits like SPI and HI, phenomic and multi-omic models captured additional variation, yielding 50–60% coincidence with adjusted phenotypic rankings for the top 30 lines. This reinforces that phenomic data can capture both genetic signals and environmentally driven trait variation within the season, providing breeders with a rapid, non-destructive tool for screening large populations when destructive biomass sampling is impractical.

Together, these results suggest that phenomic and multi-omic tools can help breeders efficiently reduce large candidate pools by selecting top-performing lines or discarding lower performers within the same season. When marker data are available, genomic prediction remains critical for refining parental choices and estimating breeding values in advance of planting, ensuring continued long-term genetic gain. Moving forward, expanding these methods to multi-location trials will be essential for testing their robustness across diverse environments. Applying this framework to other complex traits in wheat and extending it to other crops could further strengthen the practical integration of multi-omic strategies in modern breeding programs.

## 5 Conclusion

Our study demonstrates that while genomic prediction alone captures additive genetic effects and supports pre-season selection and crossing plans, it is often insufficient for complex traits like biomass partitioning. In contrast, phenomic and multi-omic models, which use in-season hyperspectral reflectance and other high-throughput data, consistently achieved higher PA and enabled non-destructive, rapid in-season selection. This allows breeders to reduce large candidate pools and focus resources on promising lines without needing destructive biomass sampling. Although multi-omic models incorporating ECs relied on the same-season data in this study, which limits pure forward prediction, historical or forecasted weather can help to approximate key environmental drivers and characterize environmental similarity when needed. Together, these approaches provide a flexible, cost-effective framework: phenomic tools screen mid-generation lines in the field, while genomic information refines parental choices and supports long-term genetic gain.

## Data Availability

The original contributions presented in the study are included in the article/**Supplementary Material**. Other data can be accessed from following repository: 10.5281/zenodo.18425150.
