# Supplementary material for: Optimizing biomass partitioning in wheat using UAV-based hyperspectral phenomic and genomic prediction: kernel-based and machine learning approaches
Source: Front Plant Sci. 2026 Feb 16;17:1740337. doi: 10.3389/fpls.2026.1740337 (PMC12950687; doi:10.3389/fpls.2026.1740337)
Supplement: Supplementary file 1 [file DataSheet1.docx]

**Journal: Frontiers in Plant Science**

**Optimizing biomass partitioning in wheat using UAV-based hyperspectral phenomic and genomic prediction: kernel-based and machine learning approaches**

Sudip Kunwar^1^, Md Ali Babar^2*^, Diego Jarquin^2^, Yiannis Ampatzidis^3^, Naeem Khan^2^, Janam Prabhat Acharya^2^, Jordan McBreen^2^, Samuel Adewale^1^, Gina Brown-Guedira^4^

^1^Plant Breeding Graduate Program, University of Florida, Gainesville, FL, 32611-0180, USA.

^2^Department of Agronomy, University of Florida, 3105 McCarty Hall B, Gainesville, FL 32608, USA.

^3^Agricultural and Biological Engineering Department, Southwest Florida Research and Education Center, University of Florida, IFAS, 2685 SR 29 North, Immokalee, FL 34142, USA

^4^Plant Science Research, USDA-ARS SEA, Raleigh, NC 27695, USA

***Correspondence:** [mababar@ufl.edu](mailto:mababar@ufl.edu)

**Supplementary Material 2**


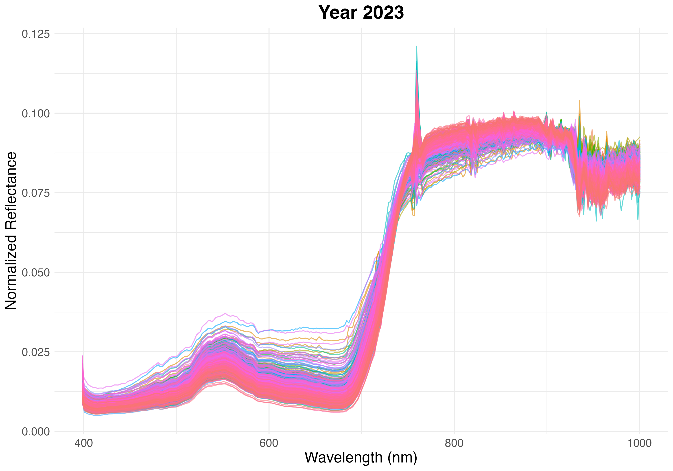

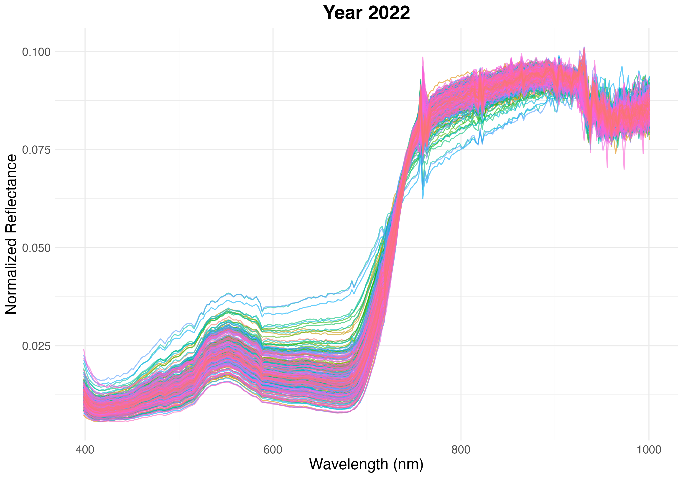


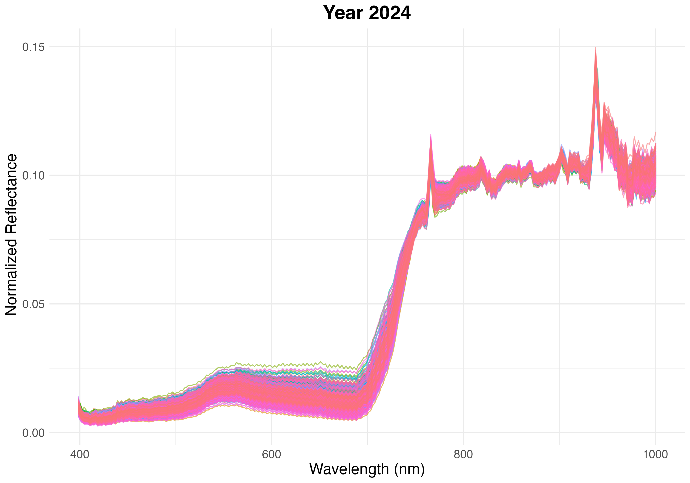

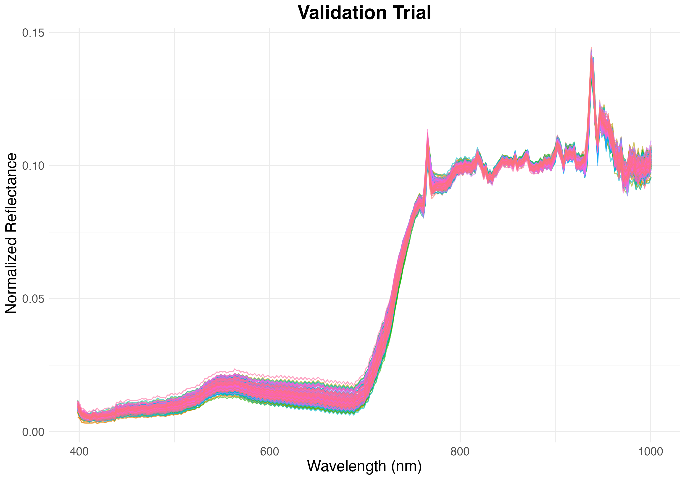


**Figure S1**. Reflectance data curve of all genotypes from all 273 bands of the hyperspectral sensor from 398 nm to 1000 nm wavelength. The reflectance data was normalized across the features to reduce the noise before constructing this curve. The data was collected from Year 2022 (top left), 2023 (top right), 2024 (bottom left), and validation trial (bottom right).


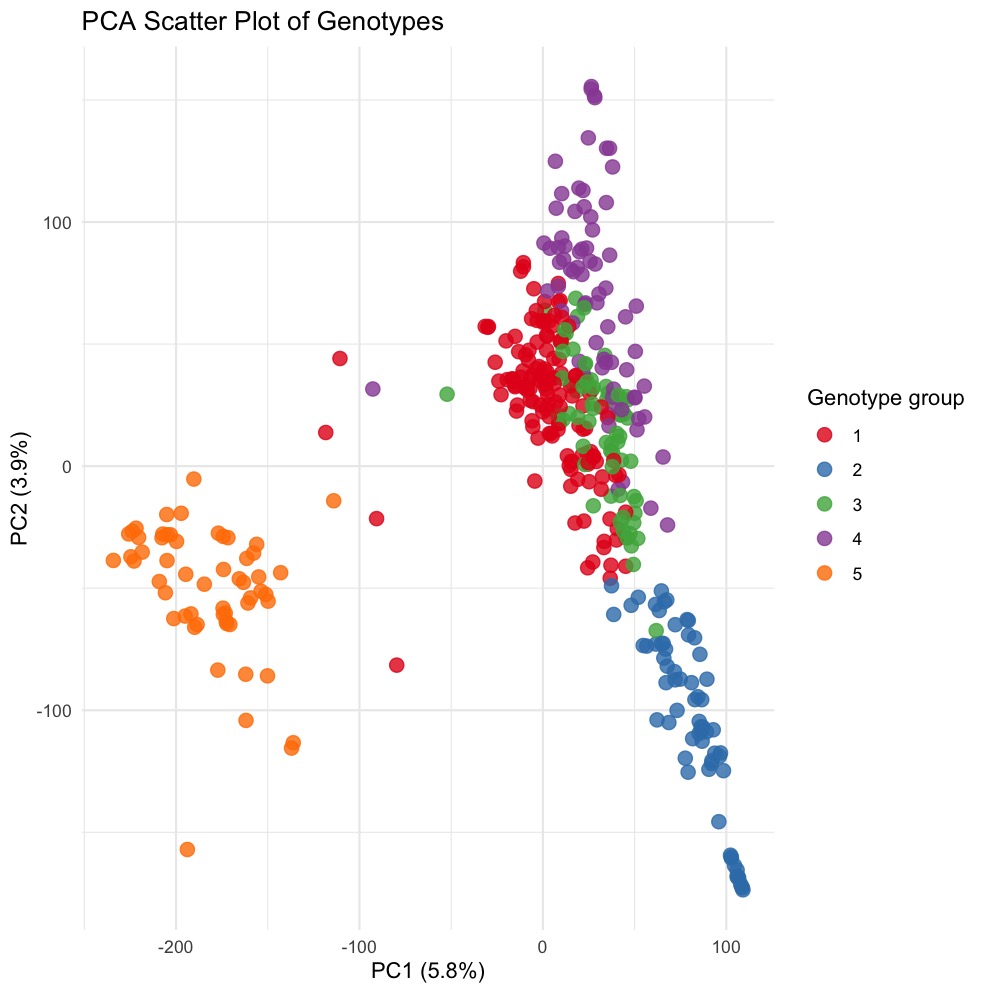


**Figure S2.** Principal component analysis (PCA) of the population structure of genotypes based on 37,395 single-nucleotide polymorphisms (SNPs) among groups inferred from principal component (PC) analysis.


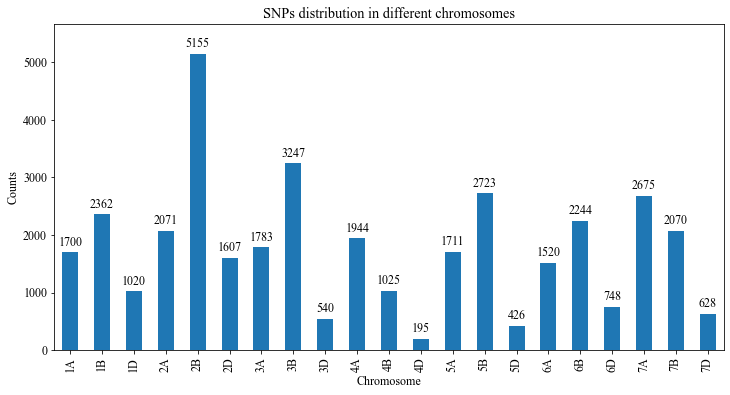


**Figure S3.** Bar diagram showing the distribution of single-nucleotide polymorphism (SNP) markers used in this study across different sub-genomes (A, B, D) and chromosomes 1-7


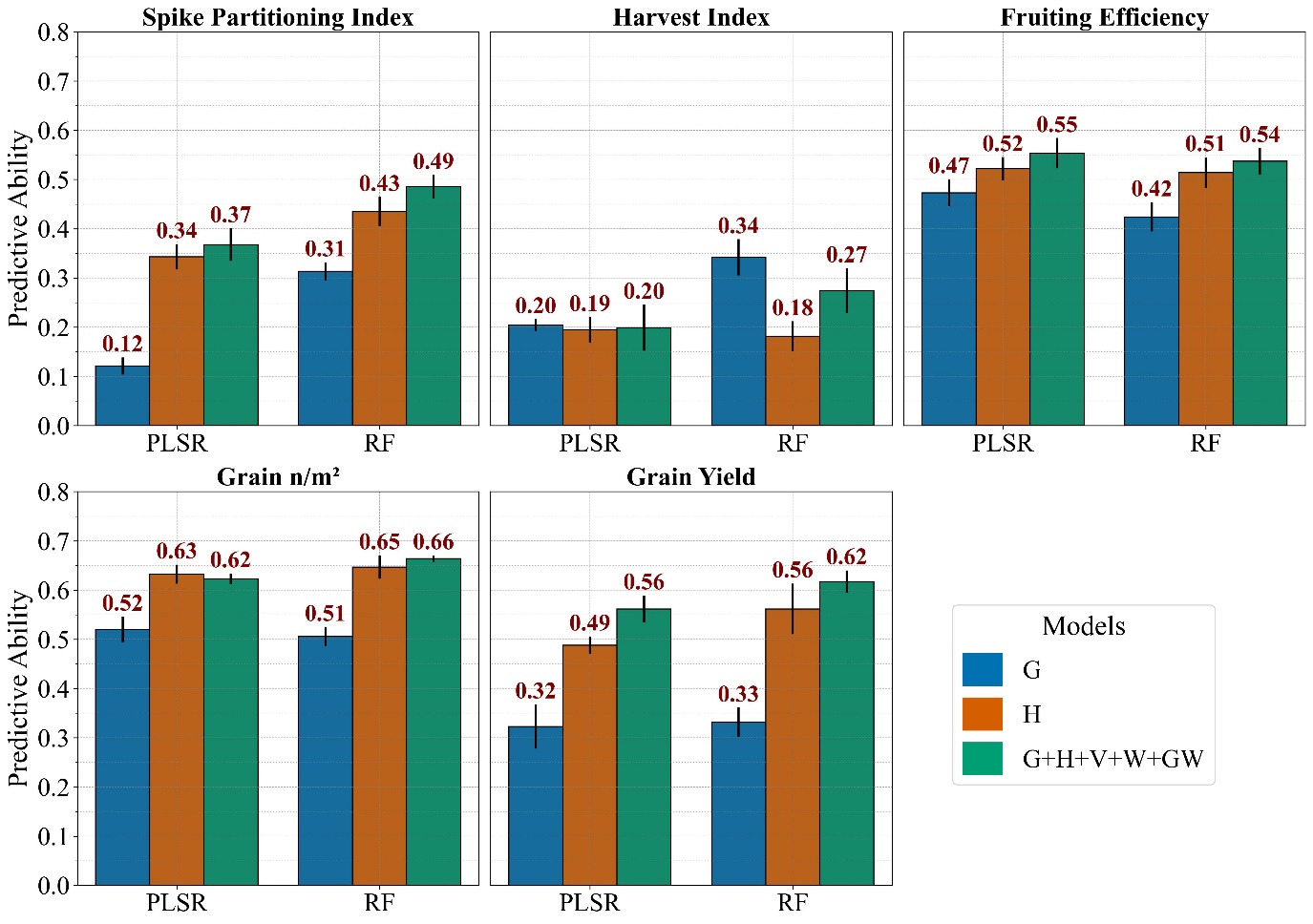


**Figure S4**. Bar diagrams showing the predictive ability of different Machine learning models to predict complex biomass partitioning and yield associated traits in a 5-fold cross-validation scheme (CV2). G, genomic prediction model; H**,** hyperspectral-based phenomic prediction model; G+H+V+W+GW, multi-omic model integrating all predictors; PLSR, partial least square regression; RF, random forest regression.


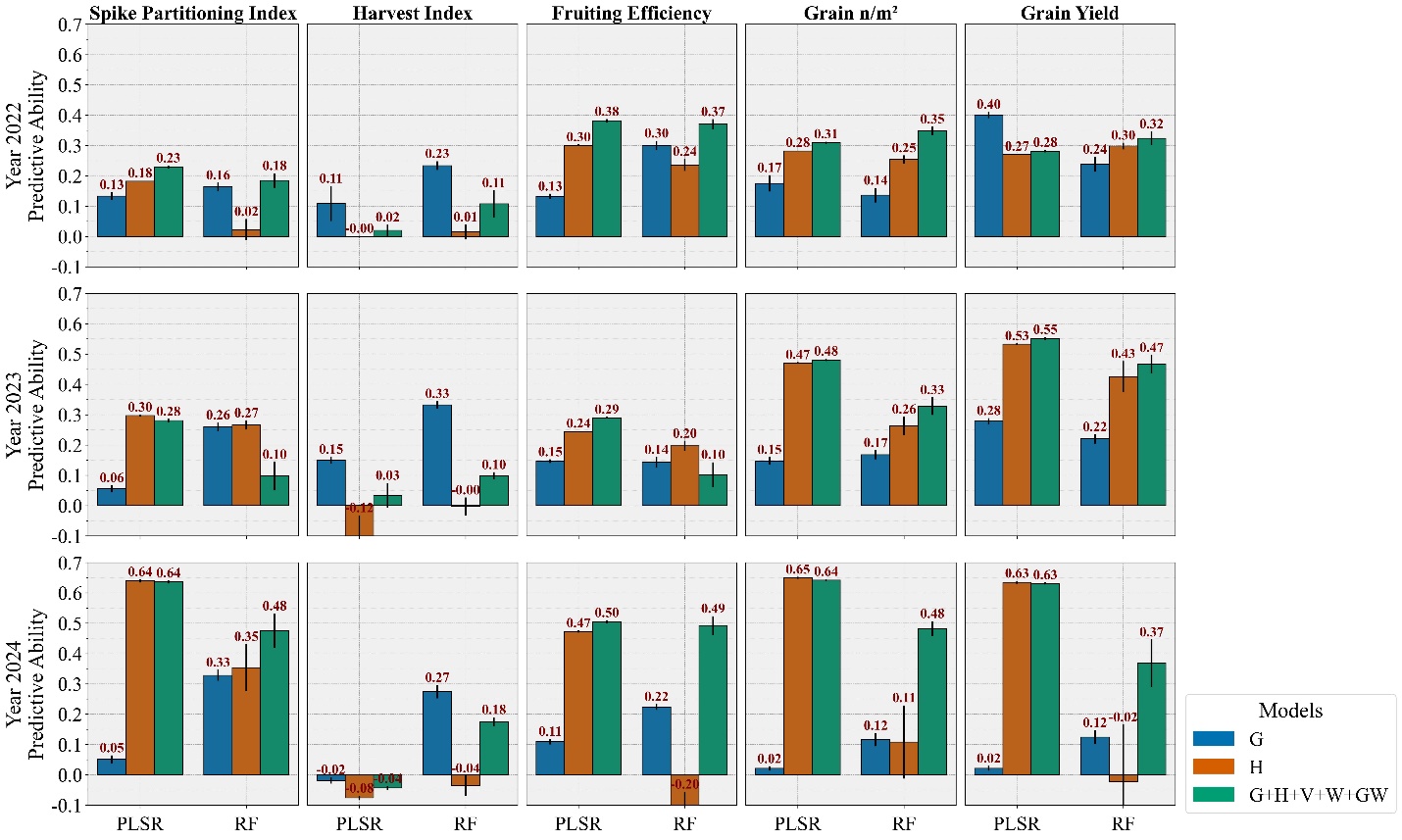


**Figure S5.** Bar diagrams showing the predictive ability of different ML models to predict phenotypic traits in leave-one-year-out (CV0). Traits for 2022 were predicted using 2023 and 2024 data, and the same was done for the other two years. G, genomic prediction model; H**,** hyperspectral-based phenomic prediction model; G+H+V+W+GW, multi-omic model integrating all predictors; PLSR, partial least square regression; RF, random forest regression.


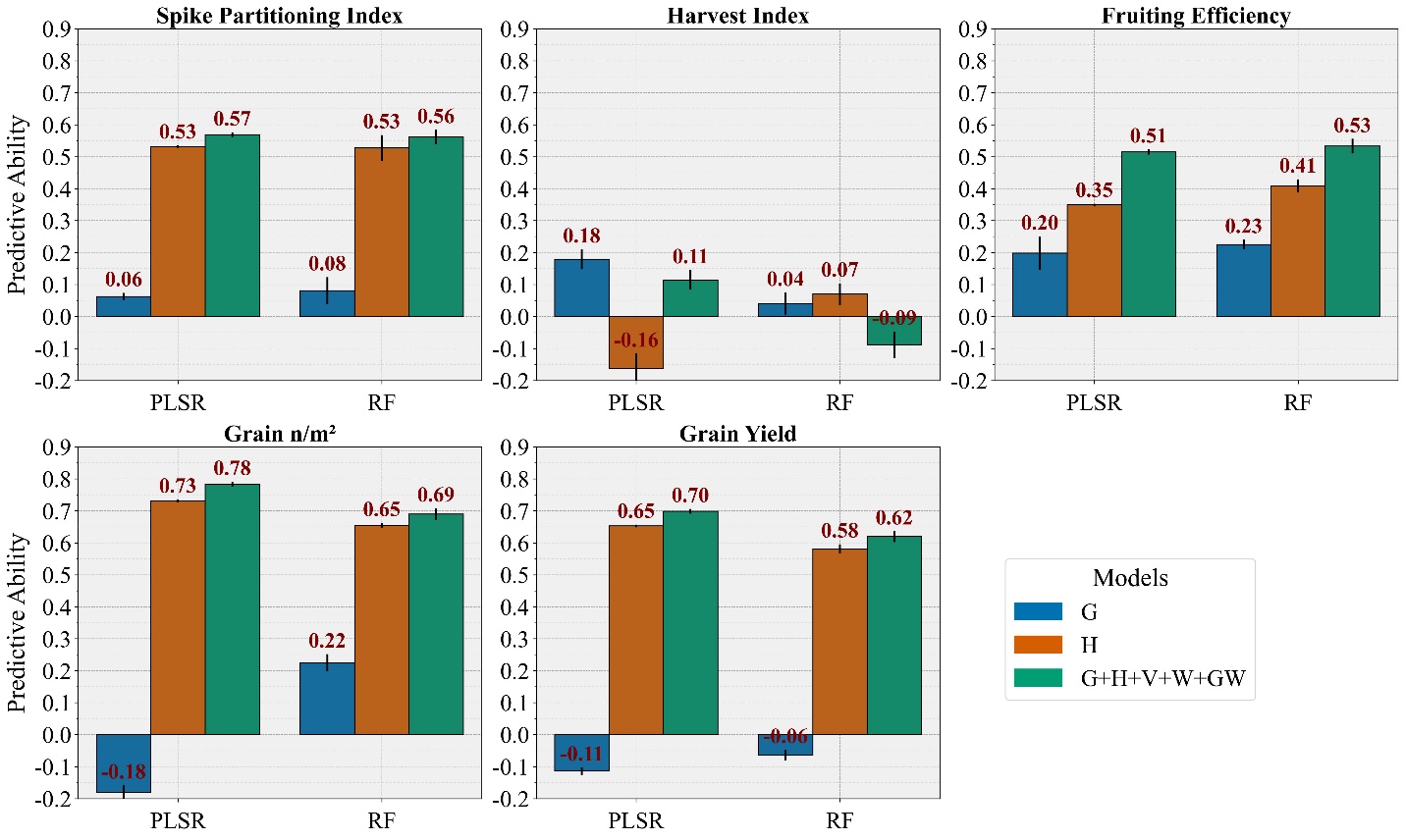


**Figure S6.** Bar diagrams showing the predictive ability of different Machine learning models to predict phenotypic traits in a separate breeding validation trial. The models were used to predict the trait in the whole validation dataset by training the model in 5-folds in the training dataset. G, genomic markers derived relationship matrix; H, hyperspectral reflectance derived relationship matrix; G+H+V+W+GW, multi-omic models combining all predictors; PLSR, partial least square regression; RF, random forest regression.
